# Genomic Integrity Safeguards Self-Renewal in Embryonic Stem Cells

**DOI:** 10.1016/j.celrep.2019.07.011

**Published:** 2019-08-06

**Authors:** Jie Su, Dandan Zhu, Zijun Huo, Julian A. Gingold, Yen-Sin Ang, Jian Tu, Ruoji Zhou, Yu Lin, Haidan Luo, Huiling Yang, Ruiying Zhao, Christoph Schaniel, Kateri A. Moore, Ihor R. Lemischka, Dung-Fang Lee

**Affiliations:** 1 Department of Cell, Developmental and Regenerative Biology, Icahn School of Medicine at Mount Sinai, New York, NY 10029, USA; 2 The Black Family Stem Cell Institute, Icahn School of Medicine at Mount Sinai, New York, NY 10029, USA; 3 The Graduate School of Biomedical Sciences, Icahn School of Medicine at Mount Sinai, New York, NY 10029, USA; 4 Cancer Biology and Genetics Program, Sloan Kettering Institute, Memorial Sloan Kettering Cancer Center, New York, NY 10065, USA; 5 Department of Integrative Biology and Pharmacology, McGovern Medical School, The University of Texas Health Science Center at Houston, Houston, TX 77030, USA; 6 Department of Endocrinology, The First Affiliated Hospital of Sun Yat-sen University, Guangzhou 510080, China; 7 Women’s Health Institute, Cleveland Clinic Foundation, Cleveland, OH 44195, USA; 8 Department of Musculoskeletal Oncology, The First Affiliated Hospital of Sun Yat-sen University, Guangzhou 510080, China; 9 The University of Texas MD Anderson Cancer Center UTHealth Graduate School of Biomedical Sciences, Houston, TX 77030, USA; 10 Institute of Clinical Pharmacology, Guangzhou University of Chinese Medicine, Guangzhou 510006, China; 11 Department of Pathophysiology, Zhongshan School of Medicine, Sun Yat-sen University, Guangzhou 510080, China; 12 Department of Pharmacological Sciences, Icahn School of Medicine at Mount Sinai, New York, NY 10029, USA; 13 Center for Stem Cell and Regenerative Medicine, The Brown Foundation Institute of Molecular Medicine for the Prevention of Human Diseases, The University of Texas Health Science Center at Houston, Houston, TX 77030, USA; 14 Center for Precision Health, School of Biomedical Informatics, The University of Texas Health Science Center at Houston, Houston, TX 77030, USA; 15 These authors contributed equally; 16 These authors contributed equally; 17 Lead Contact

## Abstract

A multitude of signals are coordinated to maintain self-renewal in embryonic stem cells (ESCs). To unravel the essential internal and external signals required for sustaining the ESC state, we expand upon a set of ESC pluripotency-associated phos-phoregulators (PRs) identified previously by short hairpin RNA (shRNA) screening. In addition to the previously described Aurka, we identify 4 additional PRs (Bub1b, Chek1, Ppm1g, and Ppp2r1b) whose depletion compromises self-renewal and leads to consequent differentiation. Global gene expression profiling and computational analyses reveal that knockdown of the 5 PRs leads to DNA damage/genome instability, activating p53 and culminating in ESC differentiation. Similarly, depletion of genome integrity-associated genes involved in DNA replication and checkpoint, mRNA processing, and Charcot-Marie-Tooth disease lead to compromise of ESC self-renewal via an increase in p53 activity. Our studies demonstrate an essential link between genomic integrity and developmental cell fate regulation in ESCs.

## In Brief

Su et al. integrate system analyses with functional genomics screening to identify five phosphoregulators (PRs) that are indispensable for ESC self-renewal. Loss of genome stability control pathways impairs ESC self-renewal, activates p53, and promotes ESC differentiation. These findings suggest that maintenance of genome integrity is essential for ESC self-renewal.

## Graphical Abstract

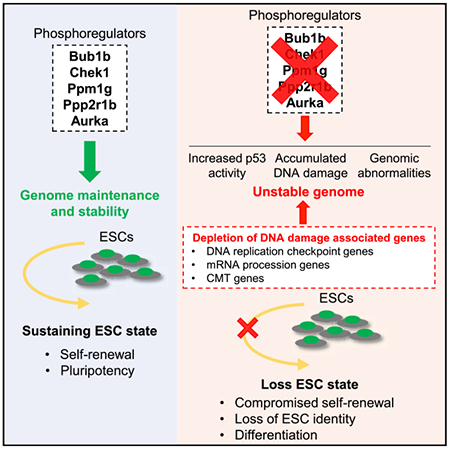

## INTRODUCTION

Embryonic stem cells (ESCs) and induced pluripotent stem cells (iPSCs) hold great promise in biomedicine as a renewable source of differentiated cells for transplantation-based therapies as well as for developing new avenues to study the etiology of diseases and develop novel diagnostic and therapeutic reagents (Gingold et al., 2016; Murry and Keller, 2008; Zhou et al., 2017). It is both urgent and essential to have a comprehensive understanding of the molecular mechanisms controlling ESC and iPSC self-renewal and the decisions governing differentiation into a wide range of mature cells to fulfill their promise in regenerative therapy and disease modeling. Recent studies primarily focused on the functions of core pluripotency transcription factors (Fidalgo et al., 2016; Gingold et al., 2014; Ivanova et al., 2006; Waghray et al., 2015), epigenetic and epitranscriptomic modifications (Aguilo et al., 2015; Ang et al., 2011; Ding et al., 2015), microRNAs (miRNAs) (Wang et al., 2013), and long noncoding RNAs (lncRNAs) (Jain et al., 2016; Luo et al., 2016) involved in maintaining the undifferentiated stem cell state. However, a global picture of cellular mechanisms controlling ESC and iPSC identity and differentiation remains incomplete.

To more globally dissect the signaling cascades required for ESC identity, we applied a short hairpin RNA (shRNA) functional genomics screening strategy of 104 ESC-associated phosphor-egulators (PRs), identifying 11 PRs with roles in maintaining ESC identity (Lee et al., 2012a, 2012b). Our earlier work investigated Aurka in greater detail and found that it controls the phosphorylation of its substrate, p53, to maintain self-renewal and the pluripotent state in a mitosis-independent manner. Although this finding provides direct evidence to support the essential role of Aurka in regulating ESC pluripotency, the role of other PRs in ESC identity has not yet been described. Here we integrate a functional genomics screen with systematic analyses to demonstrate that maintenance of genome integrity is indispensable for ESC self-renewal and pluripotency. Loss of genome stability control pathways impairs ESC self-renewal and promotes ESC differentiation. We suggest that the linkage between stem cell properties and control of genome stability is a mechanism to ensure that compromised stem cells are shuttled toward differentiation and, therefore, less capable of causing global organismal damage.

## RESULTS

### Rescue of Compromised Self-Renewal

To elucidate the essential phospho-signals required for ESC self-renewal, we built upon a previously performed shRNA functional genomics screening strategy that identified PRs whose depletion impaired ESC self-renewal at least as significantly as depletion of LIF receptor (LIFR), an essential signaling pathway in self-renewal. Our study identified Aurka as an essential kinase in maintaining ESC identity (Lee et al., 2012a). This screening study also identified 10 PRs (Acvr2a, Aurkb, Bub1b, Chek1, Dyrk3, Mapk4, Mapk13, Ppp4c, Ppm1g, and Ppp2r1b) whose depletion impaired the relative proliferation of ESCs, but their role in ESC self-renewal and differentiation has remained nebulous.

**Figure 1.**
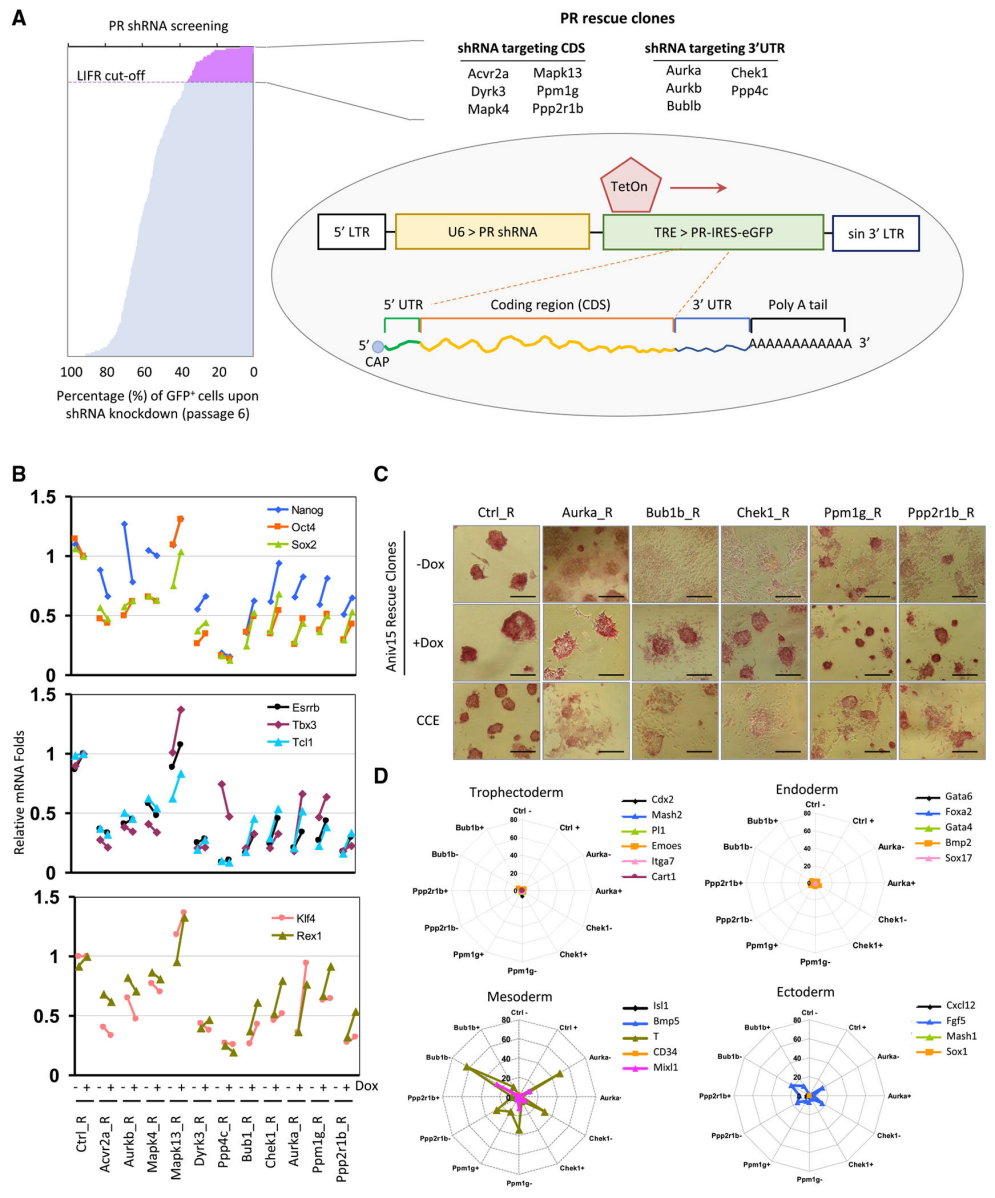
Rescue of shRNA-Induced Self-Renewal Defects by Genetic Complementation (A) A genetic complementation strategy to identify PRs involved in ESC self-renewal. (B) Defective expression of self-renewal markers is completely or partially restored in the Dox-treated Aurka_R, Bub1b_R, Chek1_R, Ppm1g_R and Ppp2r1b_R clones. n = 3 from 3 independent experiments. (C) ESC differentiation is apparent from the morphologies of the 5 PR rescue clones maintained for 5 days without Dox. This was consistent with shRNA-mediated knockdown results in CCE cells. Scale bars, 400 μm. (D) PR knockdown induces the expression of *T, Mixl1*, and *Fgf5*. The 5 PR rescue clones were maintained without feeder cells under either Dox or Dox-free conditions for 5 days. n = 3 from 3 independent experiments.

To validate these candidates and exclude potential off-target effects, we applied a genetic complementation strategy in which downregulation of an endogenous mRNA is “rescued” by doxy-cycline (Dox)-inducible expression of an shRNA “immune” exogenous version (Figure 1A) and used the Aurka rescue (Aurka_R) clone as a positive control. To eliminate shRNA-mediated knockdown of exogenous gene products, we utilized exogenous cDNA lacking the 3′ UTR to rescue shRNAs targeting the 3’ UTR; for shRNA targeting the coding region, we introduced three silent point mutations to generate a rescue cDNA lacking sequence complementarity with the shRNA. Rescue vectors lacking the shRNA target sequences were generated for Aurkb, Bub1b, Chek1, and Ppp4c, and rescue vectors containing shRNA-silent sequences were generated for Acvr2a, Dyrk3, Mapk4, Mapk13, Ppm1g, and Ppp2r1b. Lentiviral cassettes were transduced into a reverse tetracyclinetransactivator (rtTA)-expressing ESC line (Ainv15), and 3 independent rescue clones were established for each gene product. In the absence of Dox, low levels of endogenous *Nanog, Oct4, Sox2, Esrrb, Tbx3, Tcl1, Klf4,* and *Rex1* transcripts were observed in 9 rescue clone sets relative to a control (Ctrl_R) clone. However, in the presence of Dox, complete or partial pluripotent signature gene expression was restored in only 4 rescue clone sets (Bub1b_R, Chek1_R, Ppm1g_R, and Ppp2r1b_R clones) (Figures 1B and S1A; Table S1).

We found that expression of Bub1b, Chek1, Ppm1g, and Ppp2r1b (like Aurka) was higher in the Nanog_R and Esrrb_R clones in the presence of Dox (Figure S1B) as well as in primary embryonic tissues and undifferentiated ESCs (Figure S1C), further supporting the particular importance of these gene products in primitive cells. In the presence of Dox, the 4 rescue clone sets showed undifferentiated morphologies and alkaline phosphatase (AP) activity (Figures 1C and S1D). Dox-dependent SSEA1 expression was also observed (Figure S1E). Similar results were found with shRNA-transduced CCE cells (Figure 1C, bottom panels). After removal of Dox, expression levels of the primitive ectodermal marker Fgf5 and the early mesodermal markers Brachyury (T) and Mixl1, but not trophectodermal or endodermal markers, were increased (Figure 1D; Table S1), indicating that depletion of the 5 PRs promotes similar lineage-specific differentiation programs. Knockdown of the 5 PRs did not lead to significant changes in cell proliferation over 6 passages (Figure S1F), suggesting that residual PR levels are sufficient for normal proliferative function. To further measure effects on ESC self-renewal, we mixed the rescue clones with mCherry-expressing clones and co-cultured them with or without Dox. Without Dox, the mCherry^−^/mCherry^+^ ratios decreased with passage. In contrast, in the presence of Dox, the proportions of the rescue clone only decreased marginally (Figure S1G). Therefore, our results demonstrate that, in addition to Aurka, depletion of Bub1b, Chek1, Ppm1g, and Ppp2r1b impairs ESC self-renewal and promotes differentiation, confirming their essential roles in maintaining ESC identity.

### Effect on ESC Transcriptome Profiles after Downregulation of PRs

**Figure 2.**
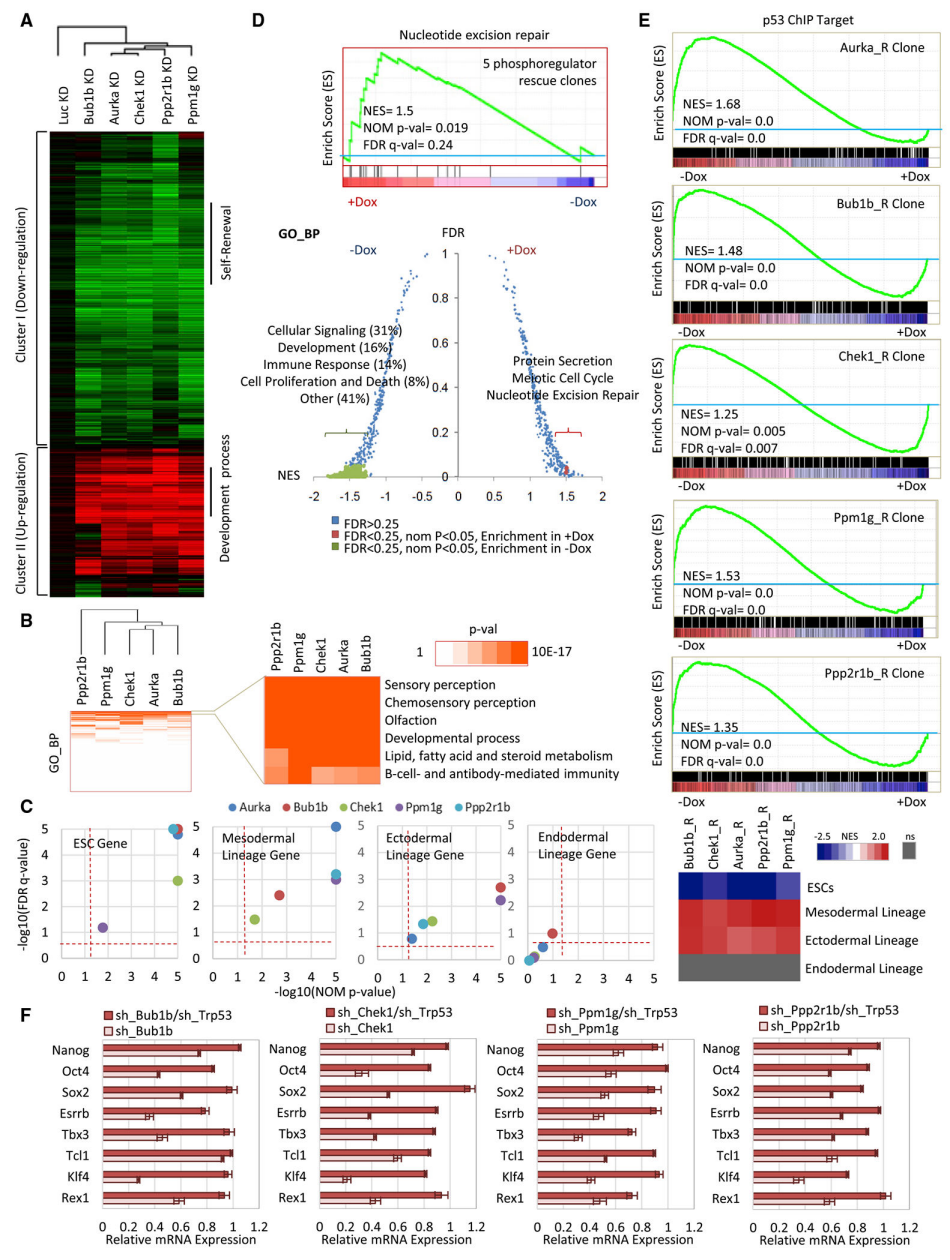
Global Transcriptome Changes after ESC Self-Renewal PR Knockdown (A) Hierarchical clustering of significant gene expression changes upon knockdown of the 5 PRs. (B) Functional categorizations of differentially expressed genes upon knockdown of the 5 PRs were analyzed by GO_BP. (C) Left panels: GSEAs of ESC, ectodermal, endodermal, and mesodermal lineage gene expression upon depletion of 5 PRs. Enriched gene sets were selected based on statistical significance (false discovery rate [FDR] q ≤ 0.25; normalized p ≤ 0.05). ns, not significant. Right panel: Heatmap of normalized enriched scores (NESs) of the lineage gene expression signatures following PR depletion. (D) GSEAs identify the enriched gene sets expressed either in ESCs with maintained expression of the 5 PRs and sustained self-renewal or after loss of these gene products and associated differentiation. A positive NES (red) represents biological processes that are enriched in the transcriptome of ESCs expressing the 5 PRs. A negative NES (green) represents biological processes that are enriched in the transcriptome of ESCs after 5 PR depletion. Enriched gene sets were selected based on statistical significance (FDR q ≤ 0.25; normalized p ≤ 0.05). Biological processes that are not significantly enriched by NES are colored in blue. (E) GSEA indicates enriched expression of p53-regulated genes (p53 chromatin immunoprecipitation [ChIP] targets) upon knockdown of the 5 PRs. (F) Knockdown of p53 completely or partially rescues the loss of expression of self-renewal genes. All values are shown as mean ± SEM with n = 3.

To provide a global picture of phosphorylation signaling maintaining ESC identity, we analyzed global gene expression changes after downregulation of each of the 5 PRs, including the Aurka_R clone for completeness. Rescue clones were maintained with or without Dox in the absence of feeder cells. After 5 days, mRNA was extracted for Illumina Beadchip microarray analyses. In the absence of Dox, we identified 602, 627, 716, 845, and 939 transcripts that were significantly changed in the Aurka_R, Bub1b_R, Chek1_R, Ppm1g_R, and Ppp2r1b_R clones, respectively, compared with the Ctrl_R clone (Table S2). We found multiple pluripotency genes in cluster I (genes downregulated upon knockdown of the 5 genes) and differentiation genes in cluster II (genes upregulated upon knockdown of the 5 genes) (Figure 2A). Hierarchical clustering based on perturbation target correlation showed overall similarities in affected targets following downregulation of the 5 PRs (Figure S2A). Mouse Gene Atlas analysis demonstrated that depletion of these PRs significantly influences genes involved in embryogenesis (Figure S2B). Panther Gene Ontology (GO) analyses of significantly altered genes revealed that knockdown of these PRs affects biological processes mainly involved in sensory perception, olfaction, and developmental processes as well as lipid, fatty acid, and steroid metabolism (Figures 2B and S2C). Consistent with our above results (Figures 1B–1D), gene set enrichment analysis (GSEA) analyses of lineage gene expression upon depletion of the 5 PRs demonstrated impaired ESC gene expression and induction of cell differentiation into mesodermal and ectodermal but not endodermal lineages (Figure 2C). To interrogate the common biological processes regulated by the 5 PRs, we grouped the 5 factor knockdown-dependent gene expression changes into a single group and investigated the alteration of GO biological processes (GO_BP) by GSEA. We examined 821 GO_BP gene sets and found 196 gene sets to be enriched following knockdown of all 5 PRs but only 3 gene sets (protein secretion, meiotic cell cycle, and nucleotide excision repair) to be enriched when the 5 PRs are expressed (Figure 2D).

Our previous study demonstrated increased expression of p53-associated mesoderm and ectoderm development genes after depletion of Aurka (Lee et al., 2012a). Interestingly, knockdown of each PR also led to increased expression of ectoderm genes and mesoderm genes (Figures 1C and 2C) as well as genes associated with DNA repair (Figure 2D). We analyzed the direct targets of p53 (Lee et al., 2010) and found increased expression levels of p53 target genes in the absence of these PRs (Figure 2E). Depletion of these PRs decreased expression of pluripotency genes and increased expression of p53-occupied ectodermal and mesodermal genes in these PR rescue cells (Figures S2D–S2F). Importantly, knockdown of p53 partially or completely rescued the loss of expression of self-renewal genes upon mRNA depletion of any of the 5 PRs (Figure 2F). These results imply a potential link between DNA damage-associated p53 signaling and PR-controlled ESC self-renewal.

### ESC Genome Stabilization by PRs

**Figure 3.**
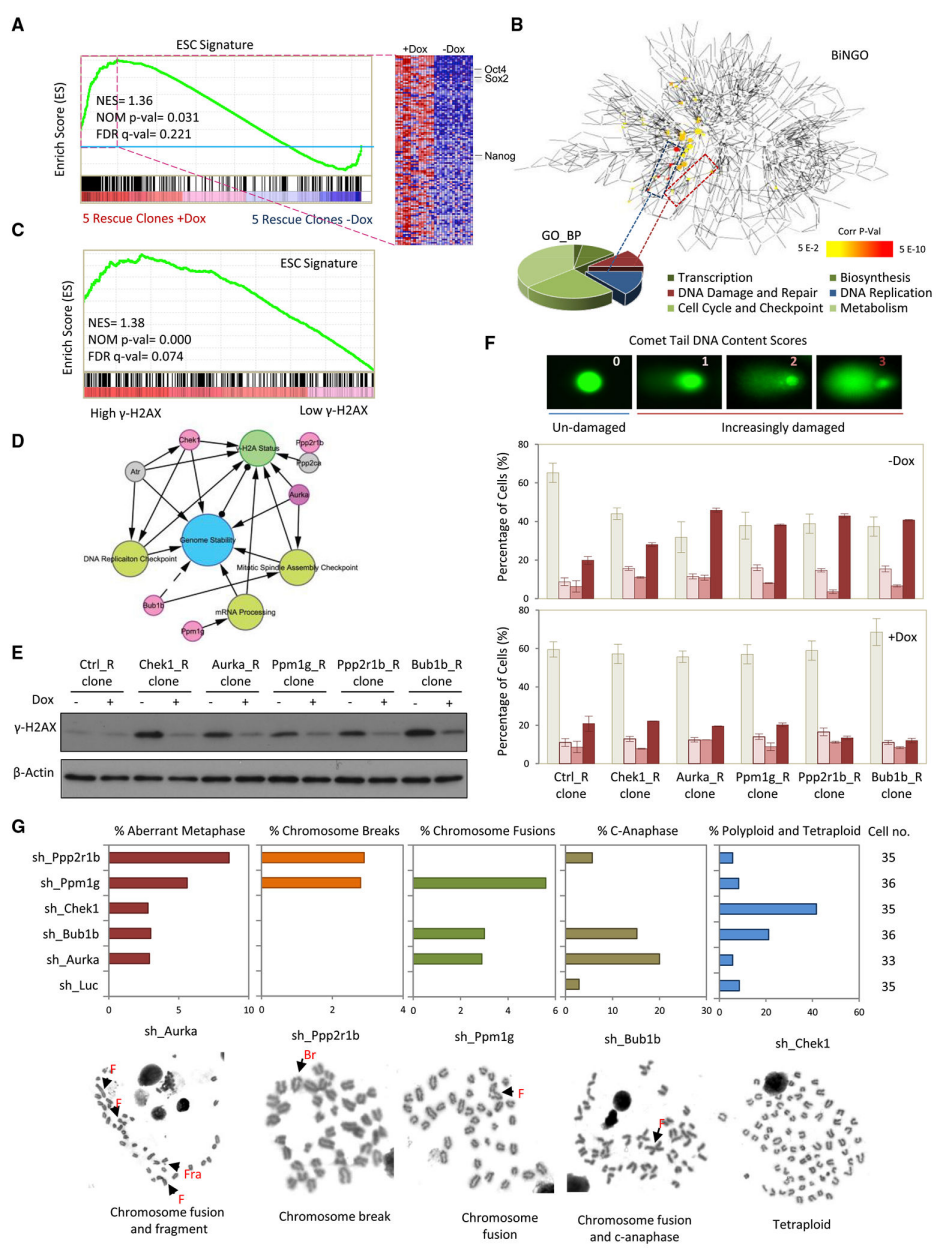
Induction of Genomic Instability upon PR Knockdown (A) GSEA comparing the transcriptomes of the 5 PR rescue clones with (−Dox) versus without (+Dox) PR depletion. (B) BiNGO analysis reveals the 119 ESC signature genes affected by PR depletion to be enriched for biological processes involved in transcription, DNA damage and repair, cell cycle and checkpoint, biosynthesis, DNA replication, and metabolism. (C) Depletion of the 119 ESC signature genes is linked to high γ-H2AX levels by GSEA. (D) Model of the potential signaling network of the 5 PRs in maintaining genome stability. Solid arrows represent known roles. Dashed arrows represent suspected roles. (E) Knockdown of the PRs increases γ-H2AX levels. The 5 PR rescue clones are maintained under feeder layer culture conditions with or without Dox for 3 days and examined by immunoblotting with an anti-γ-H2AX antibody. (F) Comet assay demonstrating an increase in DNA damage upon knockdown of the 5 PRs. Sample images from the comet assay with the different DNA content scores (0,1,2, and 3) are shown at the top. Also shown is a bar graph of the percentage of cells with each comet tail DNA content score in the PR rescue clones with and without Dox. The percentages of cells with scores of 0, 1,2, and 3 are ordered from left to right. All values are shown as mean ± SEM for n = 3. (G) Karyotype analyses reveal different levels of chromosomal abnormalities following knockdown of the 5 PRs. F, chromosome fusion; Fra, chromosome fragment; Br, chromosome breaks. Sample images of each of the 5 classes of chromosomal abnormalities are shown below.

To determine the transcriptional changes in crucial ESC-associated gene expression following Dox-mediated PR restoration, an enriched GO classification method was applied to gain further insight into biological functions. We first focused on 380 previously recognized ESC signature genes (Ben-Porath et al., 2008) and found 119 of them to be enriched following restoration of the 5 PR transcripts (Figure 3A). These genes represent the ESC-related genes consistently disrupted by loss of the 5 PRs. We then mapped the overrepresented GO_BPs of these 119 genes using the Biological Network Gene Ontology (BiNGO) tool (Maere et al., 2005) to reveal 6 major intrinsic and extrinsic signaling cascades, including metabolism, cell cycle and checkpoints, DNA replication, DNA damage and repair, biosynthesis, and transcription (Figure 3B). Impairment of ESC gene function was correlated with high γ-H2AX levels (Figure 3C), implying that the 5 PRs have roles in maintaining genome integrity. Indeed, silencing of Chek1 and Aurka increases DNA damage and genomic instability (Paulsen et al., 2009). Loss of Pp2a leads to inefficient DNA repair, resulting in hypersensitivity to DNA damage (Chowdhury et al., 2005). Moreover, Bub1b and Ppm1g modulate genome function by maintaining normal mitotic spindle assembly checkpoint function (Rao et al., 2005) and mRNA processing (Murray et al., 1999; Paulsen et al., 2009), respectively (Figure 3D).

To obtain a comprehensive picture of the relationship between the 5 PRs and DNA damage, we utilized Michigan Molecular Interactions (MiMI) (Gao et al., 2009) and identified 160 molecules hypothesized to interact with these PRs. Depletion of these 160 molecules involved in the 5 PR MiMI-generated interactome was highly associated with elevated γ-H2AX levels, implying a role of the pluripotency PR-associated interactome in regulating the DNA damage response (Figure S3A). Additionally, the expression levels of interactome components are increased in undifferentiated ESCs (Figure S3A). Network Module Identification in Cytoscape (NeMo) (Rivera et al., 2010) identified functionally related modules in this interactome and found that both anaphase-promoting complex and replication protein A complex-associated modules are enriched in undifferentiated ESCs and that depletion of these sub-interactomes is associated with high γ-H2AX levels (Figures S3B and S3C). These analyses support the concept that the 5 PRs function as a bridge linking genome integrity and ESC identity.

To investigate the roles of the 5 PRs in maintaining normal ESC genome function, we examined knockdown-dependent γ-H2AX levels. We found higher levels of γ-H2AX after each knockdown (Figure 3E). A comet assay also showed that the 5 rescue clones have similar levels of DNA damage compared with controls in the presence of the 5 PRs but that comet tail migration increased upon their depletion (Figures 3F and S3D). To further characterize whether depletion of the 5 PRs leads to genomic instability, single-cell karyotypes were examined 14 days after shRNA-mediated downregulation, revealing an increase in multiple forms of genomic abnormalities (Figure 3G). Depletion of Chek1 mainly caused polyploidy and tetraploidy, depletion of Aurka resulted in an increase in chromosome fusions and c-anaphase, depletion of Bub1b caused a broad range of genomic abnormalities, and depletion of Pppm1g and Ppp2r1b increased the percentage of aberrant metaphase and chromosome breaks and fusions. These results suggest that the 5 PRs regulate genome integrity in ESCs.

### Maintenance of Genome Stability in ESCs

**Figure 4.**
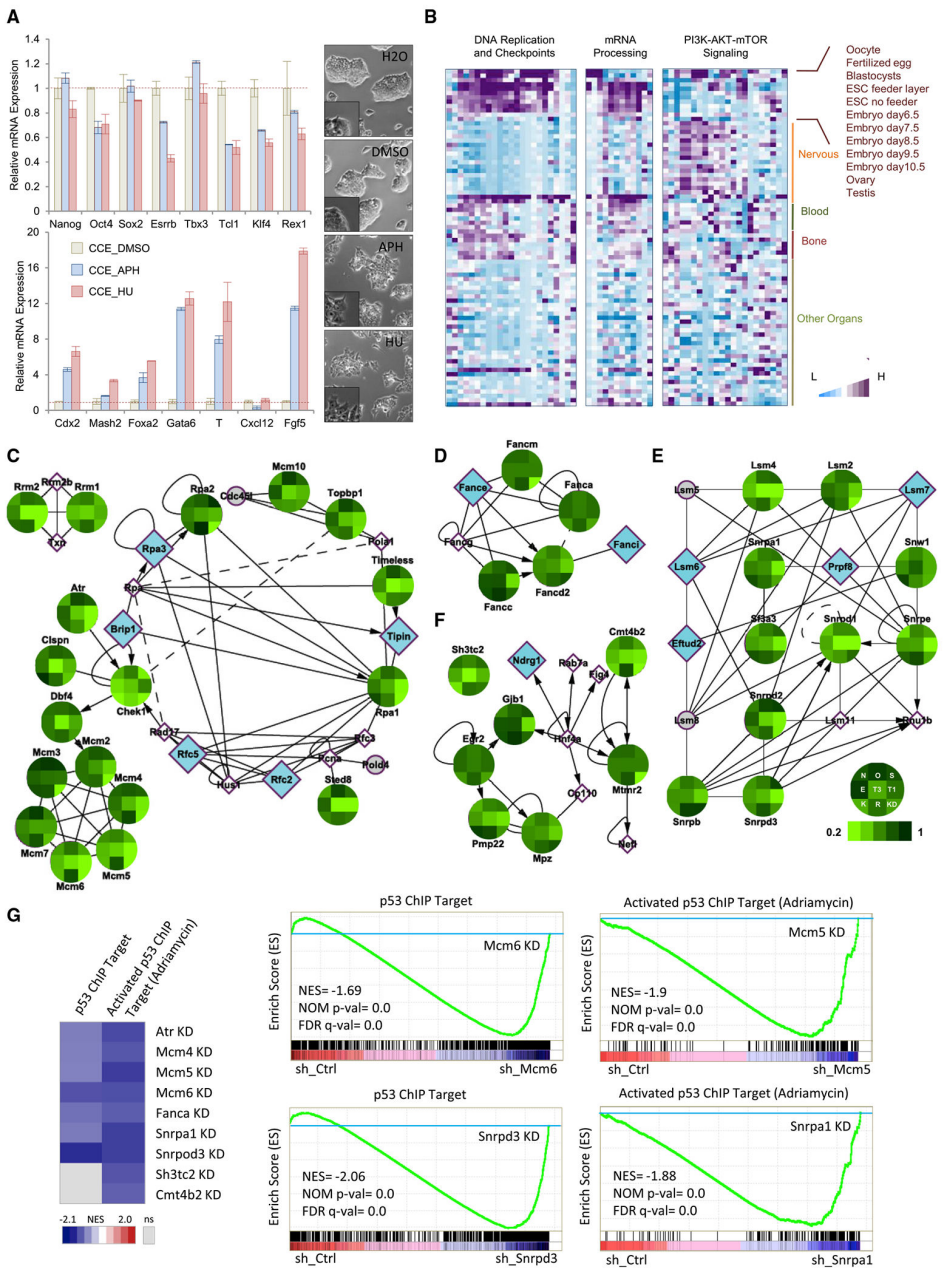
Maintenance of Genome Stability Is Essential for ESC Identity (A) The DNA damage activators HU and APH impair ESC self-renewal and promote ESC differentiation (left panels). All values shown are mean ± SEM for n = 3. Both HU- and APH-treated ESCs demonstrate smoothed colonies without defined colony boundaries (right panels). Scale bars, 100 μm. (B) Heatmaps showing that genes involved in DNA replication and checkpoint and mRNA processing, but not the phosphatidylinositol 3-kinase (PI3K)-AKT-mTOR pathway, are enriched and expressed in pre-implantation tissues, embryonic tissues, and ESCs. Gene expression data from different tissues, including the embryonic stage, nervous system, blood system, bone tissues, and other organs, were acquired from BioGPS using the Mouse Gene Atlas GNF1M. (C-F) Knockdown of genes involved in (C) DNA replication and checkpoint, (D) Fanconi anemia, (E) mRNA processing, and (F) CMT generally decreases expression of the ESC self-renewal genes *Nanog (N), Oct4 (O), Sox2 (S), Esrrb (E), Tbx3 (73), Tcl1 (T1), Klf4 (K)*, and *Rex1 (R)*, as ordered according to the legend at the bottom right. KD, knockdown efficiency of the assayed shRNA construct. The established and putative functional links between these knocked down genes are indicated by solid and dashed arrows, respectively. n = 3 from 3 independent experiments. (G) GSEA indicates enriched expression of p53-regulated genes upon knockdown of genes involved in DNA replication and checkpoint, Fanconi anemia, mRNA processing, and CMT, with p53 ChIP targets enriched in almost all knockdown (KD) shRNA constructs and activated p53 ChIP target enriched in all of the shRNA constructs (left panel). Also shown are examples of significantly enriched (FDR q ≤ 0.25; normalized p ≤ 0.05) p53-related gene sets for select shRNA constructs.

Based on our results, we hypothesized that genome integrity is essential for maintaining pluripotency. To test this directly, we treated ESCs with hydroxyurea (HU) and aphidicolin (APH), two chemicals known to induce a DNA replication stress-associated DNA damage response. Upon HU and APH treatment, ESCs dissociated from each other, lost their compact colony morphologies, and attached to the bottoms of culture dishes (Figure 4A). In addition, we observed a consistent decrease in expression levels of pluripotency transcription factors and an increase in the levels of several lineage markers (Figure 4A). These findings suggest that an increase in cellular DNA damage disrupts ESC self-renewal and triggers differentiation. It has been shown that disruption of DNA replication and checkpoints as well as mRNA processing increases DNA damage and causes genomic instability (Paulsen et al., 2009). Notably, the expression levels of molecules in these genome integrity-associated families are enriched in primary embryonic tissues and undifferentiated ESCs (Figure 4B), suggesting critical roles during embryonic development.

To more globally test whether genome integrity-associated molecules affect ESC identity, we designed shRNAs targeting 22 gene products involved in DNA replication and checkpoint-associated functions, all known to trigger DNA damage upon knockdown (Table S3). Strikingly, in 11 of 22 cases (Atr, Rpa1, Rrm1, Rrm2, Setd8, Mcm4, Mcm5, Mcm6, Mcm10, Topbp1, and Timeless), we observed detrimental effects on the expression of at least 4 of 8 pluripotency transcription factors (TFs) (Figures 4C and 4D; Table S1). shRNA-transduced ESCs exhibited lower AP activity and enhanced differentiated morphologies compared with control ESCs (Figure S4A).

In addition, suppression of several molecules essential for mRNA processing (Table S3) has been shown to result in genomic instability (Li and Manley, 2005, 2006). shRNA-mediated downregulation of 6 of 10 selected gene products (Snrpd1, Snrpd3, Snrpb, Snrpe, Sf3a3, and Snw1) resulted in decreased pluripotency transcription factor expression levels (Figure 4E; Table S1) as well as more differentiated morphologies (Figure S4A). These results strongly link increased genomic instability caused by impairment of DNA replication, checkpoint activity, and mRNA processing machineries with loss of ESC identity.

Recently, genes involved in Charcot-Marie-Tooth disease (CMT) have been reported to play roles in controlling genome stability (Paulsen et al., 2009). We studied the knockdown effect of CMT gene products on ESC self-renewal (Table S3) and found that knockdown of 4 of 7 gene products (Sh3tc2, Pmp22, Mpz, and Cmt4b2) led to substantial effects on the expression levels of at least 4 of 8 ESC pluripotency transcription factors (Figure 4F; Table S1) as well as differentiated cell morphologies (Figure S4A). These results suggest that loss of genome integrity-associated CMT gene products may substantially affect stem and progenitor cell self-renewal in CMT patients, possibly contributing to abnormal neural development and function.

We next investigated whether p53 plays a role in impairing ESC self-renewal upon depletion of DNA damage and genome instability-associated genes. We examined global transcriptome alterations following depletion of DNA replication checkpoint genes (*Atr, Mcm4, Mcm5,* and *Mcm6*), the Fanconi anemia gene (*Fanca*), mRNA processing genes (*Snrpal* and *Snrpd3*), and CMT genes (*Sh3tc2* and *Cmt4b2*). GSEA revealed enriched expression of p53-regulated genes upon knockdown of these DNA damage-associated genes (Figure 4G), indicating activation of p53 upon knockdown of these genes. Importantly, knockdown of p53 partially or completely abrogates the loss of self-renewal gene expression caused by depletion of DNA damage-associated genes (Figure S4B). These results conclusively place p53 activation at the center of the link between DNA damage/genome instability and ESC differentiation.

## DISCUSSION

Application of systematic bioinformatic analyses to the 5 PR gene products identified in our screen helped identify common signals and pathways that ultimately pointed toward maintenance of genome integrity as the unifying pathway underlying regulation of ESC identity. Mouse ESCs have been reported to have 100-fold lower mutation rates than a variety of somatic cells (Cervantes et al., 2002; Hong et al., 2007). In addition, human ESCs have an enhanced ability to repair multiple forms of DNA damage (Maynard et al., 2008). We speculate that preservation of ESC genome integrity allows maintenance of ESCs in an undifferentiated and self-renewing state and enables their organized differentiation into multiple cell types with high fidelity. Moreover, induction of differentiation in DNA-damaged pluripotent cells removes them from the highly proliferative pool, minimizing their potential to contribute to dramatic organismal damage. Genome maintenance pathways are enhanced in human ESCs, implying that their self-renewal- and pluripotency-associated functions are evolutionarily conserved. Deficiencies in genome maintenance pathways have been shown to decrease the self-renewal abilities of hematopoietic stem cells under conditions of stress (Nijniket al., 2007; Rossi et al., 2007), suggesting that other classes of stem cells may safeguard their undifferentiated properties and minimize the potential for pathological damage through similar mechanisms.

Interestingly, it is difficult to generate iPSCsfrom Fanconi anemia patient-derived cells (Raya et al., 2009). This raises the question of whether maintenance of genome stability is an essential feature of the reprogramming process. In fact, deficiencies in Atm or Trp53bp1, two proteins participating in DNA damage repair (Marión et al., 2009), lead to decreased reprogramming efficiency. Therefore, it is possible that cellular attainment of high genome integrity may function as a checkpoint during the iPSC reprogramming process and/or stabilize the acquired pluripotent state of iPSCs. Further characterization of the role of genome integrity in iPSC generation and self-renewal of iPSCs will provide additional insights into cell fate regulation and likely provide an additional lever by which the relative proportions of downstream lineages can be tweaked.

## STAR★METHODS

### LEAD CONTACT AND MATERIALS AVAILABILITY

Further information and requests for resources and reagents should be directed to and will be fulfilled by the Lead Contact, Dung-Fang Lee (dung-fang.lee@uth.tmc.edu).

### EXPERIMENTAL MODEL AND SUBJECT DETAIL

#### Mouse ESC culture

Cell culture of mouse ES cell lines CCE, E14T, and rescue clones were performed as described previously (Lee et al., 2012a, 2012b). To induce a DNA replication stress-associated DNA damage response, 1mM HU and 0.4μg/ml APH were used to treat CCE cells. Pluripotency gene expression and ESC cell morphology were examined after 24 hr treatment.

#### Derivation of Ainv15 rescue clones and differentiation assay

Generation of the PR rescue clones was described previously (Lee et al., 2012a, 2012b). Briefly, to generate rescue vectors, the PGK-Puro-IRES-eGFP fragment of the original pLKO.pig vector was replaced with a TRE-MCS-IRES-eGFP fragment (TRE: tetracycline-response element; MCS: multiple cloning site; IRES: internal ribosome entry site; eGFP: enhanced green fluorescent protein) to create a pLKO.TRE-IRES-eGFP (pLKO.tig) vector. The rescue gene was cloned and inserted in the MCS and induced in the presence of Dox. Lentivirus were generated and transduced into Ainv15 cells. Transduced cells were grown on primary MEFs in Dox-containing media and then sorted for GFP-positive cells by Fluorescent Activated Cell Sorting (FACS) on a MoFlo high speed cell sorter (Dako-Cytomation) or FACSVantage SE high speed cell sorter (BD Biosciences), and clones were expanded. Three independent clones were established for each rescue gene. For the differentiation assay, rescue clones were trypsinized and plated on tissue culture dishes for 30 min to remove primary MEFs. The media containing the floating ESCs were collected and re-plated on gelatin coated 10 cm tissue culture dishes at a concentration of 100,000 cells per dish. Cells were grown for 5 days in LIF-containing media with or without Dox and then used for AP staining and qRT-PCR.

### METHOD DETAILS

#### shRNA design, lentivirus generation, and mouse ESC transduction

shRNAs were designed by TRC library database (https://portals.broadinstitute.org/gpp/public/) and Gene Link shRNA explorer (http://www.genelink.com/sirna/shRNAi.asp) and shRNAs utilized in this study are listed in Table S3. The construction of shRNA and production of lentiviral particles were performed as described previously (Lee et al., 2012a, 2012b).

#### qRT-PCR analysis

Total RNA was extracted using Trizol (Invitrogen). 1 μg total RNA was converted into double-stranded cDNA using either the high capacity cDNA reverse transcription kit (Applied Biosystems) or the iScript cDNA synthesis kit (Bio-Rad). Quantitative PCR was performed using either the Fast SYBR Green Master Mix (Applied Biosystems) on a LightCycler480 Real-Time PCR System (Roche) or SYBR Green PCR Master Mix (Bio-Rad) on a CFX96 machine (Bio-Rad). Gene-specific primers used for this study are listed in Table S4.

#### AP and SSEA1 staining

AP and SSEA1 staining were examined using Stemgent Alkaline Phosphatase Staining Kit II (Stemgent) and Phycoerythrin conjugated mouse anti-SSEA1 antibodies (R&D Systems), respectively, following the manufacturer’s recommendations. SSEA1 marker expression was evaluated on a LSR II FACS machine (BD Biosciences).

#### Immunoblotting

Immunoblotting was performed as described previously (Hu et al., 2004; Lee et al., 2007,^2009^,2012a, 2015). Briefly, cells were lysed in RIPA-B buffer (20 mM Na_2_HPO_4_ [pH 7.4], 150 mM NaCl, 1% Triton X-100) containing protease inhibitors (1 mM phenylmethylsulfonyl fluoride, 5 mM NaF, 2 mM sodium orthovanadate, 3μg/ml aprotinin, and 750μg/ml benzamidine). The centrifuged supernatants were dissolved in protein sample buffer, resolved by SDS-PAGE, and transferred to PVDF membranes. For immunoblotting, membranes were blocked with TBST buffer (10 mM Tris-HC1 (pH 7.9), 150 mM NaC1, and 0.05% Tween 20) with either 5% skim milk or 5% BSA, incubated with anti-γH2AX (Millipore, 05-636) antibody and subsequently incubated with HRP-conjugated secondary antibodies and detected by ECL (Amersham Biosciences).

#### Microarray analysis

RNA was isolated and hybridized to Illumina Mouse WG8-V2 whole genome gene expression Beadchip microarray by Asuragen Inc., Texas. All Illumina microarray data were normalized and the differentially expressed genes were identified as described previously (Lee et al., 2012a). The heatmap was generated by the Cluster and Treeview program as described previously (Lee et al., 2009, 2012a, 2015).

#### Panther, GSEA, BiNGO and protein-protein interaction network

GO_BP analysis was performed by Panther Classification System (Thomas et al., 2003). Mouse Gene Atlas analysis was performed by Network2Canvas (Tan et al., 2013). GSEA analysis (Subramanian et al., 2005) was performed by using GSEA software with the enrichment statistic equal to weighted and the metric for ranking genes equal to signal-to-noise. GSEA for Figure 3C and S3A–S3C were performed using pre-ranked γ-H2AX status from a previous study (Paulsen et al., 2009). GSEA for Figures S3A–S3C was performed using the expression data from EB-induced mESC differentiation (Perez-Iratxeta et al., 2005). Identified gene sets from GSEA results were considered to be significantly over- or under-represented when their FDR q-value was less than 0.25 and nominal p value was less than 0.05. BiNGO analysis (Maere et al., 2005) was applied to examine GO biological processes of the enriched expression of ESC signature genes in the 5 PR (+Dox)-maintained ESC self-renewal set. The protein-protein interaction network of genome integrity-associated genes involved in DNA replication and checkpoint, mRNA processing, Fanconi anemia, and CMT disease was established by Cytoscape based on the previous publication (Paulsen et al., 2009). The DNA damage-associated genes were chosen based on previous studies. Heatmaps were generated by the Cluster and Treeview programs as described previously (Lee et al., 2009). The 5 PR-associated protein-protein interaction network was built using MiMI software (Gao et al., 2009) and sub-modules of this network were identified by NeMo (Rivera et al., 2010).

#### Comet assay

Comet assays were performed using the OxiSelect Comet Assay Kit (Cell Biolabs) following the manufacturer’s recommendations. Briefly, 750 cells were mixed with 10 volumes of melted Oxiselect Comet Agarose at 37°C and pipetted onto the Oxiselect Comet Slide. Cells were lysed with pre-chilled lysis buffer at 4°C for 1 h, incubated with pre-chilled alkaline buffer at 4°C for 30 min, and then analyzed by electrophoresis under alkaline condition. Cells were washed with pre-chilled water, fixed with 70% ethanol for 5 min, stained with Vista Green DNA dye, and visualized by Nikon ECLIPSE TE2000-U microscope with X-Cite 120 Fluorescence Illumination System. Images were captured using MetaVue Imaging (Molecular Devices). The comet tail was measured by Comet Assay IV software (Perceptive Instruments).

#### Genomic integrity analysis

Karyotype analysis was performed to examine chromosomal aberrations by the T.C. Hsu Molecular Cytogenetics Core facility at the University of Texas, M.D. Anderson Cancer Center.

### QUANTIFICATION AND STATISTICAL ANALYSIS

All experiments were performed at least three times, and all results were expressed as mean ± SEM. Multiple t test and Student’s t test were applied to determine statistical significance in the experiments. Excel and GraphPad Prism 7 were used for the statistical analysis.

### DATA AND CODE AVAILABILITY

The accession number for the microarray data reported in this paper is GEO: GSE111982. The accession number of the publicly available microarray data used in this paper are GEO: GSE23541 (Figures 2A–2C, 2E, and S2A–S2F) and GEO: GSE16428 (Figures 4G, S2E, and S2F).

## Supplementary Material

1

2

3

4

5

## Figures and Tables

**Table:** KEY REOURCES TABLE

REAGENT or RESOURCE	SOURCE	IDENTIFIER
Antibodies		
anti-γ-H2AX (1:2000 for IB)	Millipore	05-636; RRID: AB_309864
anti-β-Actin (1:2000 for IB)	Sigma	A2066; RRID: AB_476693
anti-SSEA1 (PE-conjugated) (10 μL/10^6^ cells)	R&D	FAB2155P; RRID: AB_442261
Peroxidase-AffiniPure Goat Anti-Mouse IgG	Jackson ImmunoResearch Labs	115-035-003; RRID: AB_10015289
Peroxidase-AffiniPure Goat Anti-Rabbit IgG (H+L)	Jackson ImmunoResearch Labs	111-035-003; RRID: AB_2313567
Chemicals, Peptides, and Recombinant Proteins		
Trizol	Invitrogen	15596026
hydroxyurea	Sigma	H8627
aphidicolin	Sigma	89458
ESGRO recombinant mouse LIF protein	Millipore	ESG1107
DMEM Media	GIBCO	11995073
Penicillin/Streptomycin	GIBCO	15140-122
0.25% Trypsin-EDTA	GIBCO	25200-114
Fetal Bovine Serum, Heat Inactivated	Hyclone	SH30071.03HI
Critical Commercial Assays		
OxiSelect Comet Assay Kit	Cell Biolabs	STA-351
Alkaline Phosphatase Staining Kit II	Stemgent	00-0055
High Capacity reverse transcription kit	Applied Biosystems	4368814
iScript cDNA synthesis kit	Bio-Rad	1708891
SYBR Green Master Mix	Applied Biosystems	4309155
SYBR Green PCR Master Mix	Bio-Rad	1725151
Deposited Data		
Raw and analyzed Illumina microarray data	This paper	GEO: GSE111982
Experimental Models: Cell Lines		
Mouse embryonic stem cell line CCE	Lee et al., 2012a, 2012b	N/A
Mouse Ainv15 cell line	Lee et al., 2012a, 2012b	N/A
Ctrl rescue (Ctrl_R) clone	Lee et al., 2012a, 2012b	N/A
Aurka rescue (Aurka_R) clone	Lee et al., 2012a, 2012b	N/A
Bub1b rescue (Bub1b_R) clone	This paper	N/A
Chek1 rescue (Chek1_R) clone	This paper	N/A
Ppm1g rescue (Ppm1g_R) clone	This paper	N/A
Ppp2r1b rescue (Ppp2r1b_R) clone	This paper	N/A
Oligonucleotides		
See Table S3 for oligonucleotide information of shRNAs	This paper	N/A
See Table S4 for oligonucleotide information of qPCR primers	This paper	N/A
See Table S4 for oligonucleotide information of point mutation primers	This paper	N/A
Recombinant DNA		
pLKO.pig (puro-IRES-eGFP)	Lee et al., 2012a, 2012b	N/A
pLKO.pim (puro-IRES-mCherry)	Lee et al., 2012a, 2012b	N/A
pLKO.tig Ctrl_R	Lee et al., 2012a, 2012b	N/A
pLKO.tig Aurka_3_R for generating Aurka_R clone	Lee et al., 2012a, 2012b	N/A
pLKO.tig Acvr2a_1_Rim for generating Acvr2a_R clone	This paper	N/A
pLKO.tig Aurkb_2_R for generating Aurkb_R clone	This paper	N/A
pLKO.tig Chek1_1_R for generating Chek1_R clone	This paper	N/A
pLKO.tig Mapk4_1_Rim for generating Mapk4_R clone	This paper	N/A
pLKO.tig Ppp4c_3_R for generating Ppp4c_R clone	This paper	N/A
pLKO.tig Ppm1g_2_Rim for generating Ppm1g_R clone	This paper	N/A
pLKO.tig Mapk13_2_Rim for generating Aurka_R clone	This paper	N/A
pLKO.tig Ppp2r1b_3_Rim for generating Ppp2r1b_R clone	This paper	N/A
pLKO.tig Dyrk3_2_Rim for generating Dyrk3_R clone	This paper	N/A
pLKO.tig Bub1b_1_R for generating Bub1b_R clone	This paper	N/A
Software and Algorithms		
GSEA	http://software.broadinstitute.org/gsea/index.jsp.	http://software.broadinstitute.org/gsea/index.jsp
Panther Classification System	Thomas et al., 2003	http://www.pantherdb.org/
Network2Canvas	Tan et al., 2013	http://www.maayanlab.net/N2C/#.WqwplcPwaCg
BiNGO	Maere et al., 2005	http://apps.cytoscape.org/apps/bingo
Cytoscape	Paulsen et al., 2009	https://www.cytoscape.org/
MiMI	Gao et al., 2009	http://apps.cytoscape.org/apps/mimiplugin
NeMo	Rivera et al., 2010	http://apps.cytoscape.org/apps/nemo
Cluster 3.0	Stanford University	http://bonsai.hgc.jp/~mdehoon/software/cluster/software.htm
GraphPad Prism 7	GraphPad	N/A
FlowJo	BD Biosciences	N/A
Comet Assay IV software	Perceptive Instruments	N/A
Other		
Immuno-Blot PVDF membranes	Bio-Rad	1620177

## Highlights

- Five phosphoregulators (PRs) are essential for ESC identity
- Depletion of the 5 PRs leads to activation of p53 and accumulation of DNA damage
- Depletion of genome maintenance-associated genes compromises ESC self-renewal
- Maintaining genome stability is required for ESC self-renewal
