# Supplementary material for: Genomic Integrity Safeguards Self-Renewal in Embryonic Stem Cells
Source: Cell Rep. Author manuscript; Available in PMC 2019 Aug 24. (PMC6708277; doi:10.1016/j.celrep.2019.07.011)
Supplement: 1 [file NIHMS1536801-supplement-1.pdf]

Cell Reports, Volume *28*

Supplemental Information

Genomic Integrity Safeguards

Self-Renewal in Embryonic Stem Cells

Jie Su, Dandan Zhu, Zijun Huo, Julian A. Gingold, Yen-Sin Ang, Jian Tu, Ruoji Zhou, Yu Lin, Haidan Luo, Huiling Yang, Ruiying Zhao, Christoph Schaniel, Kateri A. Moore, Ihor R. Lemischka, and Dung-Fang Lee

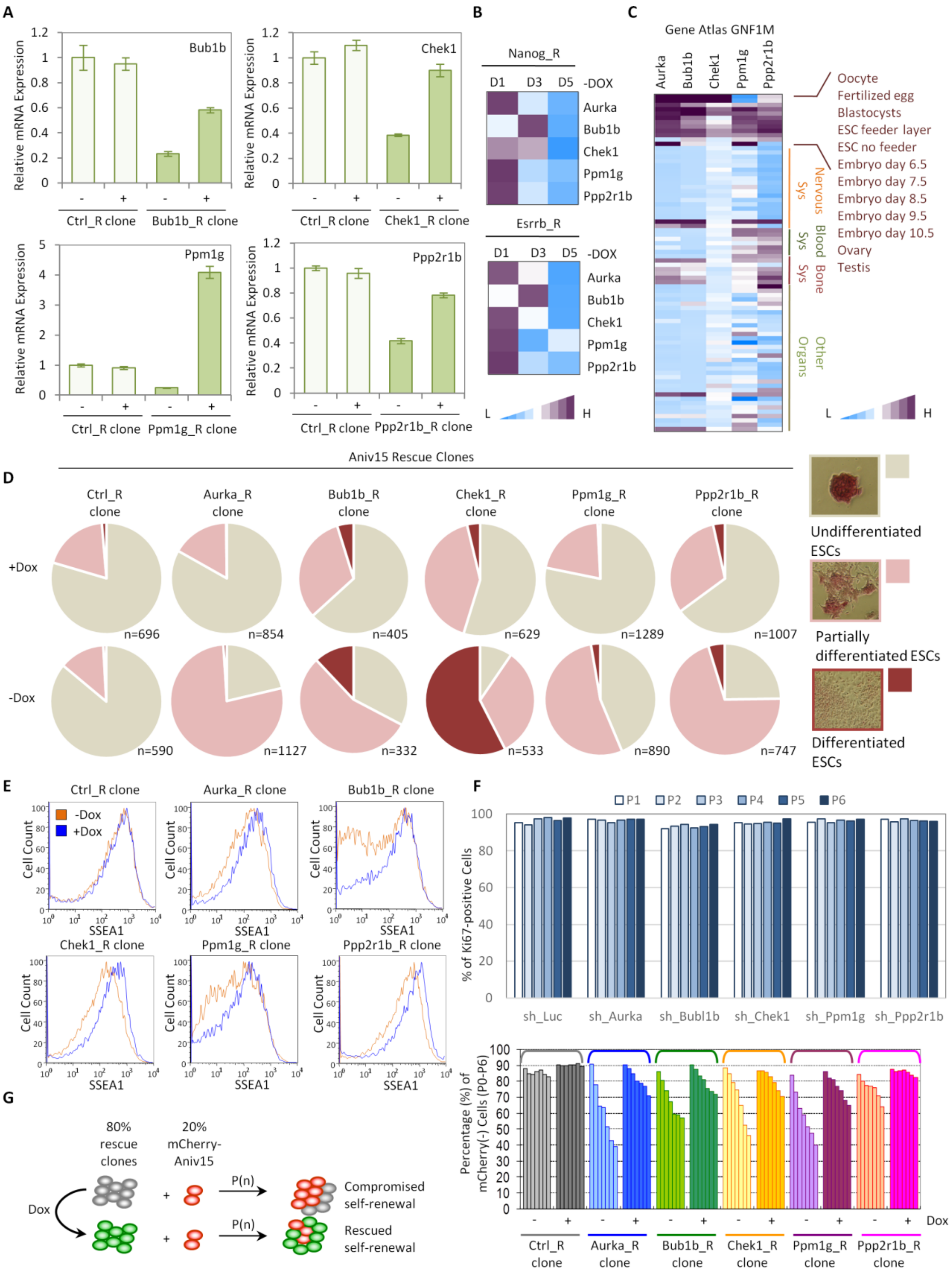
A
Relative mRNA Expression
Bub1b
Chek1
Ppm1g
Ppp2r1b
Ctrl_R clone
Bub1b_R clone
Chek1_R clone
Ppm1g_R clone
Ppp2r1b_R clone
B
Nanog_R
Esrrb_R
D1 D3 D5 -DOX
Aurka
Bub1b
Chek1
Ppm1g
Ppp2r1b
L
H
C
Gene Atlas GNF1M
Oocyte
Fertilized egg
Blastocysts
ESC feeder layer
ESC no feeder
Embryo day 6.5
Embryo day 7.5
Embryo day 8.5
Embryo day 9.5
Embryo day 10.5
Ovary
Testis
Nervous Sys
Blood Sys
Bone Sys
Other Organs
Aniv15 Rescue Clones
D
Ctrl_R clone
Aurka_R clone
Bub1b_R clone
Chek1_R clone
Ppm1g_R clone
Ppp2r1b_R clone
+Dox
-Dox
n=696
n=854
n=405
n=629
n=1289
n=1007
n=590
n=1127
n=332
n=533
n=890
n=747
Undifferentiated ESCs
Partially differentiated ESCs
Differentiated ESCs
E
Cell Count
SSEA1
-Dox
+Dox
F
P1 P2 P3 P4 P5 P6
% of Ki67-positive Cells
sh_Luc
sh_Aurka
sh_Bubl1b
sh_Chek1
sh_Ppm1g
sh_Ppp2r1b
Percentage (%) of mCherry(-) Cells (P0-P6)
Dox
G
80% rescue clones
20% mCherry-Aniv15
Dox
P(n)
Compromised self-renewal
Rescued self-renewal

Figure S1. Characterization of Bub1b_R, Chek1_R, Ppm1g_R and Ppp2r1b_R clones. Related to Figure 1.

(A) Expression of the shRNA-targeted genes in the Bub1b_R, Chek1_R, Ppm1g_R and Ppp2r1b_R rescue clones was examined in presence of Dox by qRT-PCR. All values shown are mean ± SEM for n=3.

(B) Time-course experiments demonstrate decreased expression of these 5 PRs following depletion of ESC pluripotency transcription factors Nanog or Esrrb in Nanog_R and Esrrb_R clones by withdrawal of Dox.

(C) Heat maps showing enriched expression of Bub1b, Chek1, Ppm1g and Ppp2r1b in pre-implantation tissues, embryonic tissues and ESCs. Gene expression data in different tissues including embryonic stage, nervous system, blood system, bone tissue and other organs were obtained from BioGPS using Mouse Gene Atlas GNF1M database and further analyzed by Cluster and presented by heat maps. High (H) and low (L) expression levels of indicated genes in different tissues are represented in purple and blue colors, respectively.

(D) The Bub1b_R, Chek1_R, Ppm1g_R and Ppp2r1b_R rescue clones were cultured in either Dox or Dox-free medium. After 5 days in culture, the cells were fixed and tested for AP activity. The number of undifferentiated, partially differentiated, and differentiated ESC clones were counted and represented in pie charts.

(E) Flow cytometry analyses indicate that Dox withdrawal, inducing loss of Aurka, Bub1b, Chek1, Ppm1g or Ppp2r1b, leads to lower expression of ESC surface marker SSEA1.

(F) Cell proliferation of CCE cells depleted of 5 PRs is analyzed by Ki67 staining. Transduced cells are cultured and selected by puromycin. Ki67 positive cells are analyzed at each passage from P1 to P6. n=3 from 3 independent experiments.

(G) Defective ESC self-renewal of Dox-withdrawal rescue clones. shRNA-knockdown (GFP^-) or rescued (GFP^+) cells are mixed with mCherry-expressing control cells ($mCherry^+$) in a 4-to-1 ratio, and cultured in normal ESC medium with LIF. The percentages of mCherry-negative Ctrl_R rescue cells remain similar independent of Dox. However, depletion of the 5 PRs by removal of Dox results in impaired self-renewal among rescue clones and a subsequent decline in the mCherry-negative population over 6 cell passages. n=3 from 3 independent experiments.

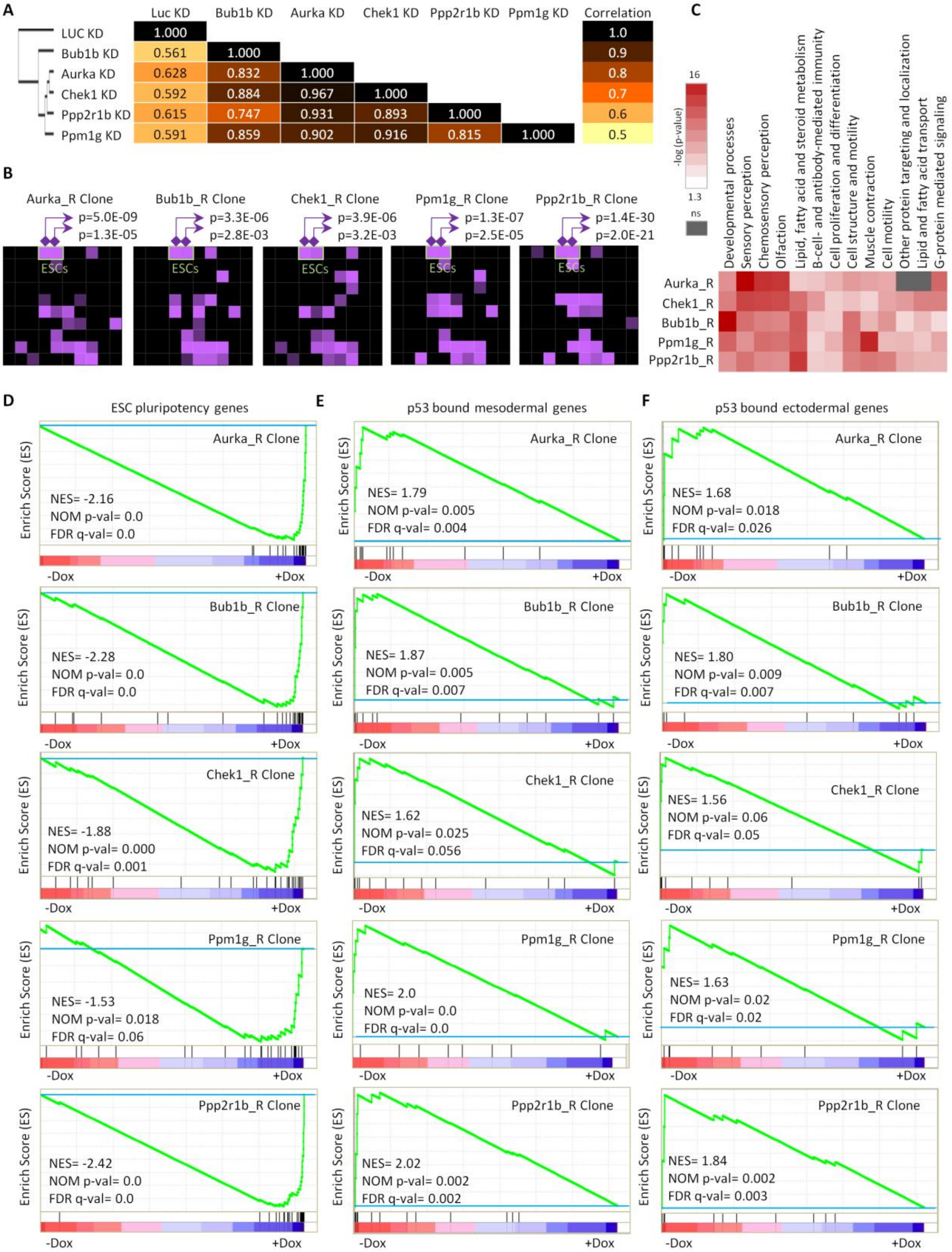
A
Luc KD
Bub1b KD
Aurka KD
Chek1 KD
Ppp2r1b KD
Ppm1g KD
Correlation
LUC KD
Bub1b KD
Aurka KD
Chek1 KD
Ppp2r1b KD
Ppm1g KD
1.000
0.561 1.000
0.628 0.832 1.000
0.592 0.884 0.967 1.000
0.615 0.747 0.931 0.893 1.000
0.591 0.859 0.902 0.916 0.815 1.000
1.0
0.9
0.8
0.7
0.6
0.5
B
Aurka_R Clone
p=5.0E-09
p=1.3E-05
Bub1b_R Clone
p=3.3E-06
p=2.8E-03
Chek1_R Clone
p=3.9E-06
p=3.2E-03
Ppm1g_R Clone
p=1.3E-07
p=2.5E-05
Ppp2r1b_R Clone
p=1.4E-30
p=2.0E-21
ESCs
C
16
-log (p-value)
1.3
ns
Developmental processes
Sensory perception
Chemosensory perception
Olfaction
Lipid, fatty acid and steroid metabolism
B-cell- and antibody-mediated immunity
Cell proliferation and differentiation
Cell structure and motility
Muscle contraction
Cell motility
Other protein targeting and localization
Lipid and fatty acid transport
G-protein mediated signaling
Aurka_R
Chek1_R
Bub1b_R
Ppm1g_R
Ppp2r1b_R
D
ESC pluripotency genes
Enrich Score (ES)
Aurka_R Clone
NES= -2.16
NOM p-val= 0.0
FDR q-val= 0.0
Bub1b_R Clone
NES= -2.28
NOM p-val= 0.0
FDR q-val= 0.0
Chek1_R Clone
NES= -1.88
NOM p-val= 0.000
FDR q-val= 0.001
Ppm1g_R Clone
NES= -1.53
NOM p-val= 0.018
FDR q-val= 0.06
Ppp2r1b_R Clone
NES= -2.42
NOM p-val= 0.0
FDR q-val= 0.0
-Dox
+Dox
E
p53 bound mesodermal genes
Aurka_R Clone
NES= 1.79
NOM p-val= 0.005
FDR q-val= 0.004
Bub1b_R Clone
NES= 1.87
NOM p-val= 0.005
FDR q-val= 0.007
Chek1_R Clone
NES= 1.62
NOM p-val= 0.025
FDR q-val= 0.056
Ppm1g_R Clone
NES= 2.0
NOM p-val= 0.0
FDR q-val= 0.0
Ppp2r1b_R Clone
NES= 2.02
NOM p-val= 0.002
FDR q-val= 0.002
F
p53 bound ectodermal genes
Aurka_R Clone
NES= 1.68
NOM p-val= 0.018
FDR q-val= 0.026
Bub1b_R Clone
NES= 1.80
NOM p-val= 0.009
FDR q-val= 0.007
Chek1_R Clone
NES= 1.56
NOM p-val= 0.06
FDR q-val= 0.05
Ppm1g_R Clone
NES= 1.63
NOM p-val= 0.02
FDR q-val= 0.02
Ppp2r1b_R Clone
NES= 1.84
NOM p-val= 0.002
FDR q-val= 0.003

Figure S2. Impairment of ESC pluripotency and induction of mesodermal and ectodermal lineage differentiation upon ESC self-renewal PR knockdown. Related to Figure 2.

(A) Hierarchical clustering of Pearson's correlation coefficient of gene expression following knockdown of the 5 PRs. ESCs depleted of the different PRs by shRNAs undergo similar patterns of gene expression, as shown by the high correlation scores off the diagonal in all but the Luc knockdown cells. KD indicates knockdown.

(B) Enrichment analysis using Network2Canvas of significantly influenced genes following depletion of these 5 PRs includes genes identified as enriched in ESCs using Mouse Gene Atlas data (small boxed region), implying an essential role of these 5 PRs in embryo development.

(C) Panther classification analyses of significantly altered genes reveal that knockdown of these 5 PRs affects biological processes mainly involved in sensory perception, olfaction, developmental processes, as well as lipid, fatty acid and steroid metabolism. ns indicates not significant.

(D) GSEA analyses indicate downregulation of ESC pluripotency genes upon knockdown of Aurka, Bub1b, Chek1, Ppm1g and Ppp2r1b.

(E-F) GSEA analyses reveal upregulation of p53-regulated mesoderm and ectoderm target genes upon knockdown of Aurka, Bub1b, Chek1, Ppm1g and Ppp2r1b.

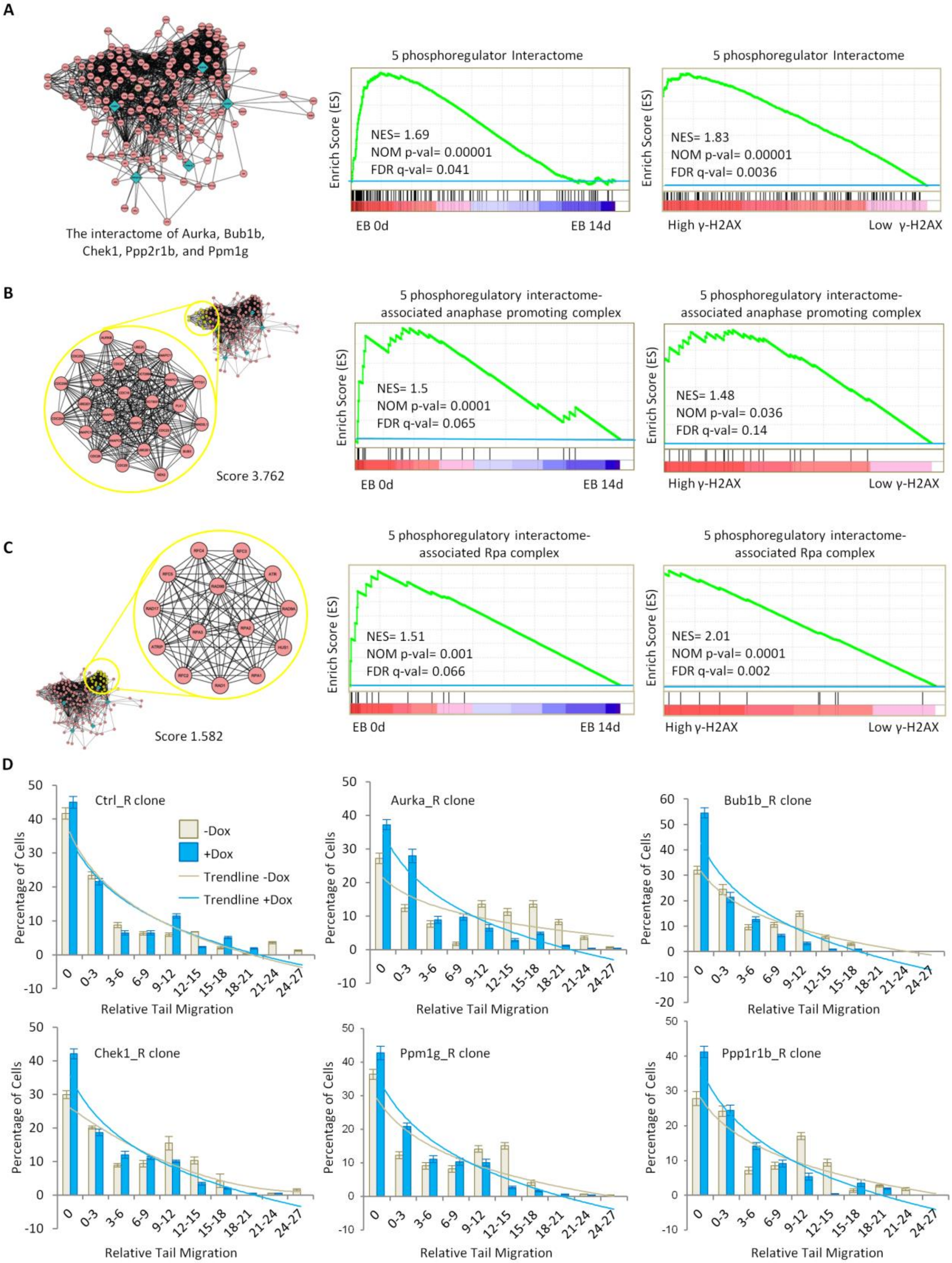
A
The interactome of Aurka, Bub1b, Chek1, Ppp2r1b, and Ppm1g
5 phosphoregulator Interactome
Enrich Score (ES)
NES= 1.69
NOM p-val= 0.00001
FDR q-val= 0.041
EB 0d
EB 14d
5 phosphoregulator Interactome
Enrich Score (ES)
NES= 1.83
NOM p-val= 0.00001
FDR q-val= 0.0036
High γ-H2AX
Low γ-H2AX
B
Score 3.762
5 phosphoregulatory interactome-associated anaphase promoting complex
Enrich Score (ES)
NES= 1.5
NOM p-val= 0.0001
FDR q-val= 0.065
EB 0d
EB 14d
5 phosphoregulatory interactome-associated anaphase promoting complex
Enrich Score (ES)
NES= 1.48
NOM p-val= 0.036
FDR q-val= 0.14
High γ-H2AX
Low γ-H2AX
C
Score 1.582
5 phosphoregulatory interactome-associated Rpa complex
Enrich Score (ES)
NES= 1.51
NOM p-val= 0.001
FDR q-val= 0.066
EB 0d
EB 14d
5 phosphoregulatory interactome-associated Rpa complex
Enrich Score (ES)
NES= 2.01
NOM p-val= 0.0001
FDR q-val= 0.002
High γ-H2AX
Low γ-H2AX
D
Ctrl_R clone
-Dox
+Dox
Trendline -Dox
Trendline +Dox
Aurka_R clone
Bub1b_R clone
Chek1_R clone
Ppm1g_R clone
Ppp1r1b_R clone
Percentage of Cells
Relative Tail Migration
0
0-3
3-6
6-9
9-12
12-15
15-18
18-21
21-24
24-27

Figure S3. Maintenance of genome integrity by 5 PRs and their associated interactome. Related to Figure 3.

(A) Expression of genes from the 5 PR-associated interactome (left panel) is enriched in undifferentiated ESCs (EB 0d) compared with ESCs allowed to differentiate (EB 14d) (middle panel) and also in conditions of increased γ-H2AX (right panel) by GSEA analyses.

(B-C) Both Apc- and Rpa-associated functional modules are identified in the 5 PR-associated interactome by NeMo analysis. GSEA analyses reveal enriched expression of these two modules in undifferentiated ESCs (EB 0d) as well as in cells with high γ-H2AX levels.

(D) Knockdown of 5 PRs leads to increased comet tail lengths. Relative comet tail migration was examined and calculated by the Comet Assay IV program. X-axis represents the relative length of comet tails. All values shown are mean ± SEM for n=3.

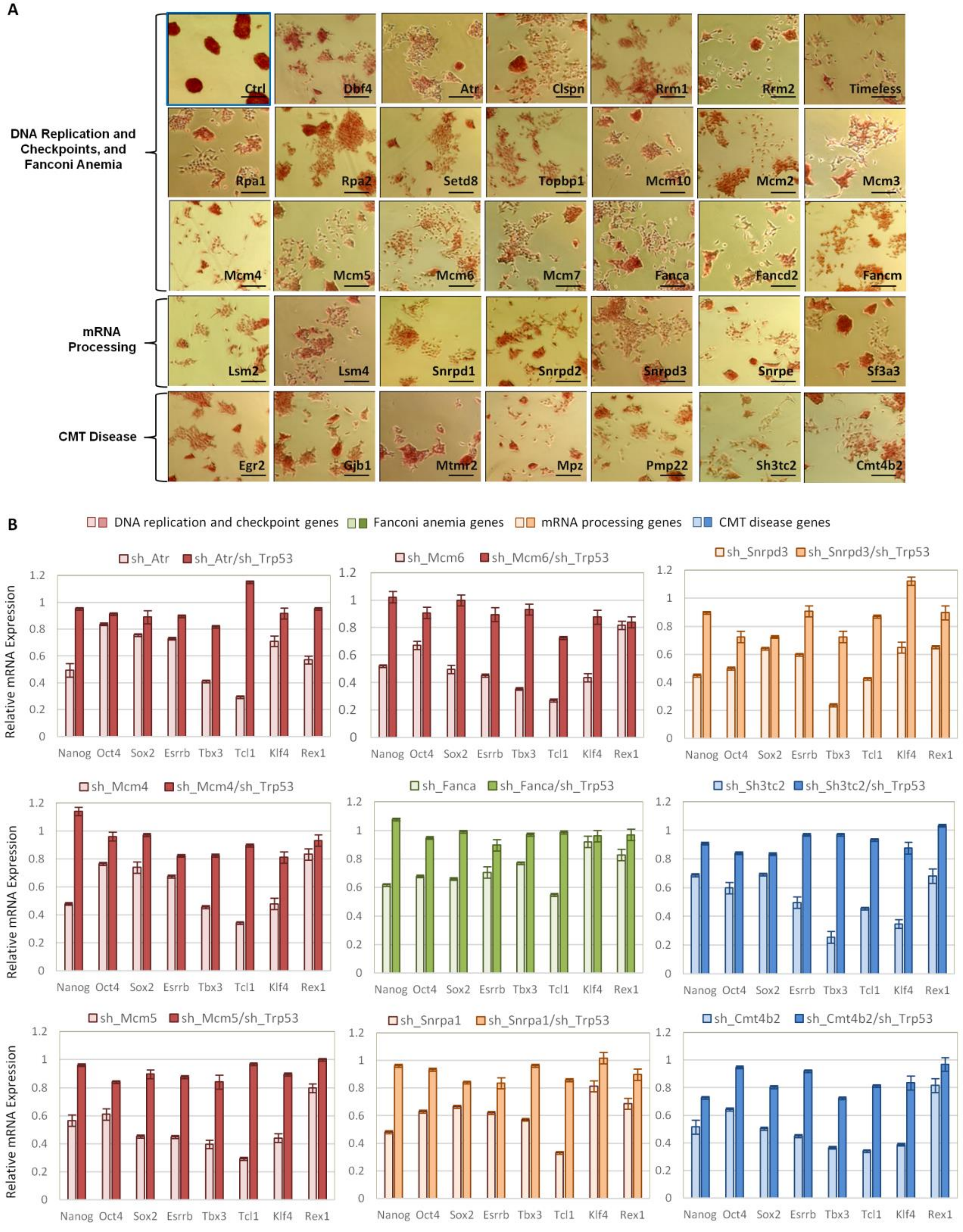
A
DNA Replication and Checkpoints, and Fanconi Anemia
mRNA Processing
CMT Disease
Ctrl
Dbf4
Atr
Clspn
Rrm1
Rrm2
Timeless
Rpa1
Rpa2
Setd8
Topbp1
Mcm10
Mcm2
Mcm3
Mcm4
Mcm5
Mcm6
Mcm7
Fanca
Fancd2
Fancm
Lsm2
Lsm4
Snrpd1
Snrpd2
Snrpd3
Snrpe
Sf3a3
Egr2
Gjb1
Mtmr2
Mpz
Pmp22
Sh3tc2
Cmt4b2
B
DNA replication and checkpoint genes
Fanconi anemia genes
mRNA processing genes
CMT disease genes
sh_Atr
sh_Atr/sh_Trp53
sh_Mcm6
sh_Mcm6/sh_Trp53
sh_Snrpd3
sh_Snrpd3/sh_Trp53
sh_Mcm4
sh_Mcm4/sh_Trp53
sh_Fanca
sh_Fanca/sh_Trp53
sh_Sh3tc2
sh_Sh3tc2/sh_Trp53
sh_Mcm5
sh_Mcm5/sh_Trp53
sh_Snrpa1
sh_Snrpa1/sh_Trp53
sh_Cmt4b2
sh_Cmt4b2/sh_Trp53
Relative mRNA Expression
0
0.2
0.4
0.6
0.8
1
1.2
Nanog
Oct4
Sox2
Esrrb
Tbx3
Tcl1
Klf4
Rex1

Figure S4. Effect of depletion of genes involved in DNA replication and checkpoint, Fanconi anemia, mRNA processing and CMT on pluripotency transcription factor gene expression. Related to Figure 4.

(A) ESC differentiation is apparent from the morphologies of ESC-expressing shRNAs targeting genes involved in DNA damage when examined by AP staining. Scale bar, 400 μm.

(B) Depletion of p53 attenuates the loss of expression of self-renewal genes *Oct4, Sox2, Nanog*, *Esrrb*, *Tcl1*, *Tbx3*, *Klf4*, and *Rex1*. All values shown are mean ± SEM for n=3.
